# Insulin resistance mediates high-fat diet-induced pulmonary fibrosis and airway hyperresponsiveness through the TGF-β1 pathway

**DOI:** 10.1038/s12276-019-0258-7

**Published:** 2019-05-27

**Authors:** Yoon Hee Park, Eun Yi Oh, Heejae Han, Misuk Yang, Hye Jung Park, Kyung Hee Park, Jae-Hyun Lee, Jung-Won Park

**Affiliations:** 1 Institute for Allergy, Yonsei University College of Medicine, Seoul, Korea; 2 Department of Internal Medicine and Gangnam Severance Hospital, Yonsei University College of Medicine, Seoul, Korea; 3 Department of Internal Medicine, Yonsei University College of Medicine, Seoul, Korea

**Keywords:** Immunology, Experimental models of disease

## Abstract

Prior studies have reported the presence of lung fibrosis and enhanced airway hyperresponsiveness (AHR) in mice with high-fat-diet (HFD)-induced obesity. This study evaluated the role of TGF-β1 in HFD-induced AHR and lung fibrosis in a murine model. We generated HFD-induced obesity mice and performed glucose and insulin tolerance tests. HFD mice with or without ovalbumin sensitization and challenge were also treated with an anti-TGF-β1 neutralizing antibody. AHR to methacholine, inflammatory cells in the bronchoalveolar lavage fluid (BALF), and histological features were evaluated. Insulin was intranasally administered to normal diet (ND) mice, and in vitro insulin stimulation of BEAS-2b cells was performed. HFD-induced obesity mice had increased insulin resistance, enhanced AHR, peribronchial and perivascular fibrosis, and increased numbers of macrophages in the BALF. However, they did not have meaningful eosinophilic or neutrophilic inflammation in the lungs compared with ND mice. The HFD enhanced TGF-β1 expression in the bronchial epithelium, but we found no differences in the expression of interleukin (IL)−4 or IL-5 in lung homogenates. Administration of the anti-TGF-β1 antibody attenuated HFD-induced AHR and lung fibrosis. It also attenuated goblet cell hyperplasia, but did not affect the AHR and inflammatory cell infiltration induced by OVA challenge. The intranasal administration of insulin enhanced TGF-β1 expression in the bronchial epithelium and lung fibrosis. Stimulating BEAS-2b cells with insulin also increased TGF-β1 production by 24 h. We concluded that HFD-induced obesity-associated insulin resistance enhances TGF-β1 expression in the bronchial epithelium, which may play an important role in the development of lung fibrosis and AHR in obesity.

## Obesity: A trigger for asthma onset

Insulin resistance may be an important causative factor underlying the increased risk of asthma and other respiratory issues in obese individuals. Obesity doubles the likelihood of developing asthma, with symptoms that are more difficult to control than in non-obese patients. The connection between these conditions is poorly understood, but researchers led by Jung-Won Park, Yonsei University College of Medicine, Seoul, South Korea, have identified a potential mechanism. They demonstrated that a signaling molecule called TGF-β1 contributes to airway sensitivity and tissue scarring in a mouse model of diet-induced obesity. Subsequent experiments showed that treatment with insulin also gives rise to increased TGF-β1 production in the mouse lung. Since insulin resistance is a common feature of obesity, resulting in abnormally high levels of circulating insulin, this could also account for the increased risk of respiratory problems.

## Introduction

In the last decade, the number of diagnosed asthma patients has increased to over 300 million people worldwide^1^. Recent studies have demonstrated that obesity is an important causative factor of asthma, and that the risk of asthma is doubled in obese patients compared with normal weight patients^2,3^. Furthermore, a decrease in body weight improves asthma outcomes^4^. As the proportion of people with obesity is steadily increasing, the burden of obesity on asthma is also becoming increasingly important^5^. Obesity-associated asthma differs from none obesity-associated asthma in several ways, specifically, patients with obesity-associated asthma struggle with poor asthma control and are frequently resistant to conventional treatments^6,7^.

However, the mechanism underlying the association between obesity and asthma remains controversial. Obesity itself is associated with increases in the levels of various systemic proinflammatory mediators, such as C-reactive protein (CRP), leptin, and interleukin (IL)−6^8–10^. High-fat-diet (HFD)-induced obesity may also induce increased levels of IL-1β, tumor necrosis factor (TNF)-α, IL-17, and transforming growth factor (TGF)-β in lung tissue^11–14^. We previously demonstrated that a HFD is significantly associated with AHR and lung fibrosis in mice with TNF-α-producing macrophages, but not with enhanced accumulation of eosinophils or neutrophils in the lung tissue, and the enhancement in AHR was eliminated by treating the obese mice with exercise^4,8,11^. The association between AHR and lung fibrosis without allergic inflammation in HFD-induced obesity indicates a causal relationship between airway remodeling and the development of AHR in this model.

TGF-β, an adipokine, has diverse roles in the maintenance of cellular homeostasis, lung development, and physiology^15^. TGF-β stimulates the production of connective tissue by fibroblasts, and enhanced expression of TGF-β in the lungs may induce tissue dysfunctions, such as the lung fibrosis and airway remodeling seen in asthma and chronic bronchitis^15–17^. TGF-β can be secreted by epithelial cells, fibroblasts, eosinophils, mast cells, and Treg cells^17^. Several cytokines and chemokines related to allergic inflammation, such as IL-13, adenosine, VEGF, and CCR2, stimulate the production of TGF-β1 from these cells^18^. However, in addition to the TGF-β1 pathway, other mechanisms, such as the IL-13 and periostin pathway, also engage in the development of subepithelial fibrosis in the lungs and airway remodeling of asthma^19^. Recent studies have shown that TGF-β1 expression is also enhanced by HFD-induced obesity in mice^12,13,20^ and Drosophila^21^. Furthermore, insulin resistance is a critical problem in obesity and is associated with proinflammatory reactions involving various immune cells and cytokines^22^. Some studies have reported that TGF-β is associated with insulin resistance^21,23^, and that blocking TGF-β signaling protects against the development of HFD-induced obesity and diabetes in mice^24^. In vitro experiments have demonstrated that cells from insulin-resistant subjects and bronchial cells treated with insulin exhibit increased TGF-β activation and subsequently increased lung fibrosis^25,26^. These findings suggest that TGF-β1 might be the connection between airway remodeling and AHR in obesity-associated asthma^16^. However, the role of TGF-β1 may be complicated as some investigators have also reported that TGF-β1 has anti-inflammatory effects on allergic asthma^27^.

In this study, we hypothesized that the insulin resistance or increased insulin levels in obesity might play a significant role in obesity-induced airway hyperresponsiveness and lung fibrosis via the TGF-β1 pathway. Therefore, we evaluated whether insulin resistance and TGF-β1 production affect HFD-induced AHR and lung fibrosis using a murine model.

## Materials and methods

### Animals

**Fig. 1:**
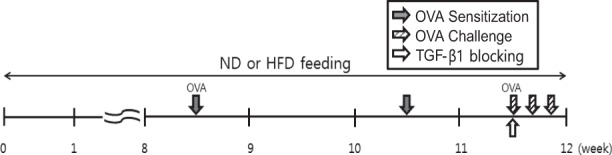
Study scheme. ND normal diet, HFD high-fat-diet, OVA ovalbumin

Male C57BL/6 mice were purchased from Japan-SLC (Hamamatsu, Japan). The study scheme for this study is shown in Fig. 1, and was approved by the Institutional Animal Care and Use Committee of Yonsei University College of Medicine (IACUC number: 2017-0349). Each group consisted of four mice, and all mice were maintained under standard conditions (room temperature of 21–24 °C, relative humidity of 45–70%, and a 12-h light/dark cycle) at conventional animal facilities, which were fully accredited by the Association for Assessment and Accreditation of Laboratory Animal Care International.

### Feeding

All mice were fed a normal diet (ND) or HFD for 12 weeks. The ND (D12450B; Research Diets Inc., New Brunswick, NJ, USA) contained 10% kcal from fat, and the HFD (D12492; Research Diets Inc.) contained 60% kcal from fat. The mice were weighed weekly.

### Ovalbumin-induced asthma model

Mice were sensitized with a mixture containing 20 µg of ovalbumin (OVA; InvivoGen, San Diego, CA, USA) and Imject Alum (100 µL per mouse; Thermo Fisher Scientific, Rockford, IL, USA) by intraperitoneal (i.p.) injection on days 0 and 14. One week after the last injection, the mice were challenged with OVA (30 µg per mouse) via intranasal injection on three consecutive days. For intranasal administration, the mice were anesthetized with isoflurane (*jw* Pharmaceutical, Seoul, Korea). Two days after the last OVA challenge, the mice were killed for analysis (Fig. 1).

### Glucose and insulin tolerance tests and Insulin measurement

An oral glucose tolerance test (OGTT) and insulin tolerance test (ITT) were performed at the 10th and 11th weeks, respectively, after HFD feeding. Food was withheld from the mice overnight for the OGTT and withheld for 4 h for the ITT. The OGTT was performed by orally administering glucose (2 g/kg body weight; Sigma-Aldrich, St. Louis, MO, USA). For the ITT, the mice were administered insulin (2 U/kg body weight; Sigma-Aldrich) by i.p. injection. The blood glucose level was measured in blood collected from the tail vein at 0, 30, 60, 90, and 120 min after glucose or insulin injection using blood glucose strips and an Accu-Check glucometer (Roche, Mannheim, Germany). Mouse insulin concentrations were measured after 12-h fasting and feeding states using an ELISA kit (Alpco, Salem, NH, USA).

### Anti-TGF-β1-blocking antibody studies

To block TGF-β1, a rabbit anti-mouse TGF-β neutralizing mAb (100 µg/mouse; R&D Systems Inc., Minneapolis, MN, USA) or rabbit IgG antibody (100 µg/mouse; R&D Systems) was administered once via the intravenous (i.v.) route 4 h before the first OVA challenge (Fig. 1).

### Measurement of methacholine airway hyperresponsiveness

Mice were anesthetized with pentobarbital sodium (50 mg/kg; Hanlim Pharma Co., Seoul, Korea) by i.p. injection at 48 h after the last challenge. An 18-gauge cannula was inserted into the anesthetized mice via tracheostomy, and then the mice were connected to a ventilator. AHR in response to various concentrations of inhaled aerosolized methacholine (MCh) (6.25, 12.5, 25.0, 50.0, and 100.0 mg/ml; Sigma-Aldrich) was measured using a forced oscillation technique (FlexiVent^®^ 5.1; SCIREQ, Montreal, Canada).

### Collection and processing of the bronchoalveolar lavage fluid

To collect the bronchoalveolar lavage fluid (BALF), the lungs were irrigated with 1 ml of HBSS (Thermo Fisher Scientific, Waltham, MA, USA) through a tracheal tube. The total number of cells was counted using a hemocytometer. The collected BALF was centrifuged for 3 min at 10,000 rpm and 4 °C. The whole cells were resuspended in HBSS, and BALF cell smears were prepared by cytocentrifugation (Cytospin 3, Thermo Fisher Scientific, Billerica, MA, USA). The cytocentrifuged slides were stained with Leukostat (Fisher Diagnostics, Fair Lawn, NJ, USA), and at least 200 inflammatory cells were counted.

### Enzyme-linked immunosorbent assay and lung homogenates

To analyze cytokine levels, the right lung tissue was homogenized with 50 mg/ml tissue protein extraction buffer (Thermo Fisher Scientific, Rockford, IL, USA) using a tissue homogenizer (Biospec Products, Bartlesville, OK, USA). After an incubation for 30 min on ice, the homogenates were centrifuged at 1000 *g* for 10 min. The supernatants of the lung homogenates were collected, passed through a 0.45-micron filter (Gelman Science, Ann Arbor, MI, USA) and stored at −80 °C until cytokine levels were measured. The measured cytokine levels were normalized to the lung tissue weight. Concentrations of IL-4, IL-5, and TGF-β1 in the lung homogenates or cell supernatants were measured by ELISA (R&D Systems, San Diego, CA, USA) according to the manufacturer’s instructions.

### Histological analysis

The left lung of mice was fixed in 4% formalin and embedded in paraffin. Lung sections were cut (3–4 -µm thickness) and stained with hematoxylin and eosin (H&E) for general examination, periodic acid-Schiff (PAS) to measure goblet cell hyperplasia, and Masson’s trichrome (MT) to assess fibrosis. The tissue sections were examined using an Olympus BX40 microscope in conjunction with an Olympus U-TV0.63XC digital camera (Olympus BX53F, Center Valley, PA, USA). Images were acquired using cellSens Standard 1.6 imaging software. The H&E- and PAS-stained sections were scored using a semiquantitative system. The severity of inflammation was scored as follows: 0, no inflammatory cell infiltrates; 1, minimal inflammation; 2, mild inflammation; 3, moderate inflammation; and 4, robust inflammation. Goblet cell hyperplasia was measured by the number of PAS-positive cells per 1000 airway epithelial cells. A quantitative analysis of fibrosis was conducted using Metamorph^®^ (Molecular Devices, Sunnyvale, CA, USA). The fibrotic area was assessed by measuring the color-pixel count over the preset threshold color for the entire field containing several bronchial tubes in MT-stained slides at ×200 magnification.

### Anti-TGF-β1 immunohistochemistry

Paraffin blocks were cut into 4-µm sections, deparaffinized, and rehydrated in xylene and ethanol solutions. Antigen retrieval was performed with the tissue sections in a microwave oven using sodium citrate buffer (pH 6.0) for 10 min. The sections were incubated overnight at 4 °C with an anti-TGF-β antibody (Ab92486, Abcam, Cambridge, UK), followed by incubation with appropriate biotinylated secondary antibodies (PK-6101, 6105, or 6200; Vector Laboratories, Burlingame, CA, USA). The immunocomplexes were visualized via 3,3′-diaminobenzidine (SK-4100; Vector Laboratories) staining.

### Hydroxyproline analysis

To measure the collagen content of the lungs, a hydroxyproline assay was performed using a commercial hydroxyproline assay kit (Cell Biolabs Inc., San Diego, CA, USA). Lung tissue was homogenized in PBS. The homogenates were added to an HCl solution and incubated for 3 h at 120 °C. The hydrolysates were centrifuged at 10,000 *g* for 3 min and transferred to 96-well plates for analysis. Hydroxyproline levels were assessed with a spectrophotometer at 560 nm.

### In vivo and in vitro bronchial epithelial cell stimulation with insulin

We intranasally administered 20 µg of insulin once daily to C57BL/6 mice for 11 days and killed the mice 24 h after the last administration. Human bronchial epithelial BEAS-2B cells were grown and cultured. The BEAS-2B cells were maintained in bronchoepithelial basal medium (Lonza, Basel, Switzerland) with supplements in six-well plates. After reaching 80% confluence, the cells were treated with two concentrations of insulin (0.1, 1, and 10 µg/ml) for 1, 4, and 24 h. To measure the total TGF-β1 content, the cell supernatant was acidified with acetic acid and then neutralized.

### Statistical analysis

All results are expressed as the mean ± SEM. One-way analysis of variance (ANOVA) was performed using SPSS statistical software version 12.0 (SPSS Inc., Chicago, IL, USA). The AHR data were analyzed with repeated-measures ANOVA followed by a post hoc Bonferroni test. The other data were analyzed with one-way ANOVA followed by a post hoc Bonferroni test. *P* < 0.05 was considered statistically significant.

## Results

### HFD-induced changes in body weight, oral glucose tolerance, insulin tolerance, and insulin concentration

**Fig. 2:**
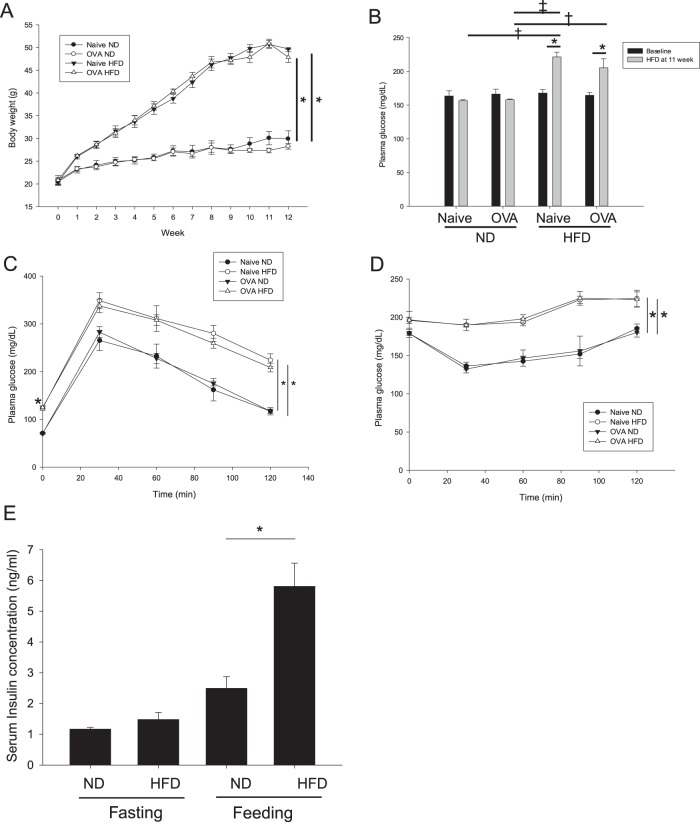
Physiologic and metabolic changes of studied groups. Comparisons of body weight (**a**), blood glucose levels (**b**), the results of an oral glucose tolerance test (**c**) performed during the 10th week of HFD feeding, and an insulin tolerance test performed during the 11th week of HFD feeding (**d**) between HFD mice and ND mice. Serum insulin concentrations were measured during the 12th week of HFD feeding. ^†^*P* *<* 0.05; ^‡^*P* < 0.01; *P < 0.001. HFD high-fat-diet, ND normal diet

Body weight in the HFD groups (naive HFD and OVA-challenged HFD) began to increase from the first week of HFD feeding. At killing during the 12th week, the mean weight of the mice in the ND groups was 29.2 g, whereas that of the mice in the HFD groups was 47.4 g (Fig. 2a). The ND and HFD groups displayed the same blood glucose level at baseline. At the 11th week, the blood glucose levels of the HFD groups were significantly higher than those of the ND groups (Fig. 2b). At 10 weeks of HFD feeding, the mice in the HFD groups exhibited significantly impaired glucose tolerance (Fig. 2c) compared with those in the ND groups. The next week, the insulin tolerance test was performed, and we found that the HFD groups had impaired insulin tolerance (Fig. 2d). Mouse serum insulin concentrations after 12-h fasting and feeding states were also measured during the 12th week of HFD feeding. Fasting insulin levels were not different between the ND and HFD groups, but in the feeding state, the insulin concentration of the HFD groups was significantly higher than that of the ND groups (Fig. 2e).

### AHR to MCh, BALF, expression of cytokines, and lung histological features of HFD mice

**Fig. 3:**
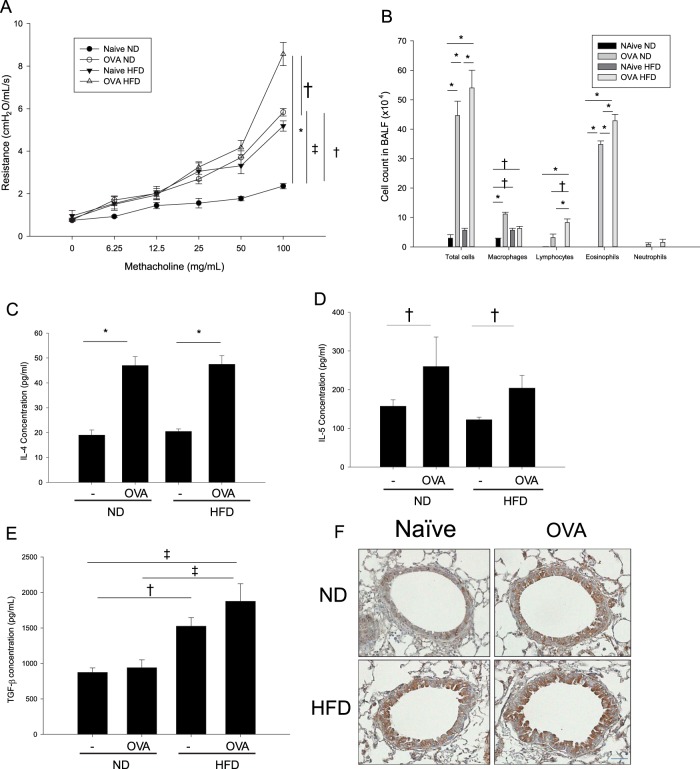
AHR and inflammatory features of naive HFD and OVA-HFD models. AHR to MCh (**a**), inflammatory cell profile in the BALF (**b**), IL-4 (**c**) and IL-5 levels (**d**), and TGF-β1 (**e**) concentrations in lung homogenates. Immunohistochemical staining for TGF-β1 (F) in tissue from naïve HFD, OVA-HFD, naive ND, and OVA-ND mice are shown. ^†^*P* < 0.05; ^‡^*P* < 0.01; *P < 0.001. AHR airway hyperresponsiveness, MCh methacholine, BALF bronchoalveolar lavage fluid, HFD high-fat-diet, IL interleukin, OVA ovalbumin, ND normal diet, TGF transforming growth factor

We next examined AHR and performed BALF cell analysis with HFD mice. The AHR to MCh in naive HFD mice was significantly higher than that in naive ND mice (Fig. 3a). BALF analysis revealed that the HFD did not induce the infiltration of eosinophils, neutrophils, or lymphocytes, but did increase the number of macrophages. The HFD also did not exacerbate allergic inflammation in the BALF of mice with OVA-induced asthma (Fig. 3b). ND mice with OVA-induced asthma had high-expression levels of IL-4 and IL-5, but naive HFD mice did not express significantly higher IL-4 or IL-5 levels in the lung tissue than naive ND mice (Fig. 3c, d). In addition, the OVA-HFD group did not exhibit enhanced IL-4, IL-5, or TGF-β1 expression compared with the OVA-ND group. Instead, the naive HFD group exhibited enhanced TGF-β1 expression in lung homogenates (Fig. 3e) compared with the naive ND group, and immunohistochemical staining showed increased expression of TGF-β1 in naive HFD mice, especially in the bronchial epithelium (Fig. 3f). We further investigated whether OVA-induced asthma affected the expression of TGF-β1 in the lungs of mice but found that OVA challenge did not affect the protein expression of TGF-β1 in the bronchial epithelium (Fig. 3f).

**Fig. 4:**
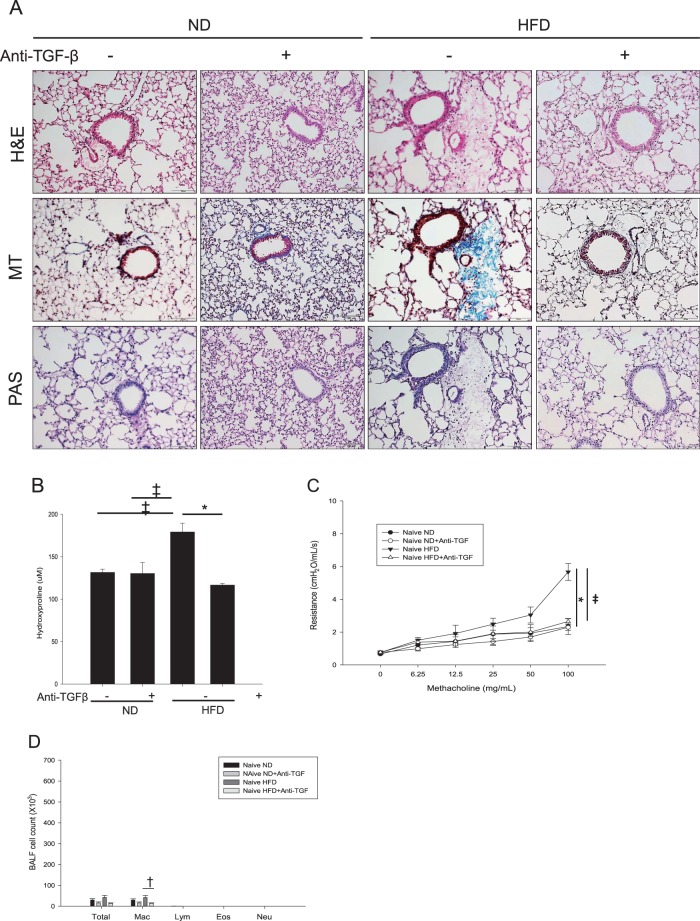
Histological features of the lungs from HFD-induced obesity mice given anti-TGF-β mAb treatment. H&E, PAS, and MT staining features (**a**) are shown at ×200 magnification. The collagen content of the lung tissue was measured by a hydroxyproline assay (**b**). Panels 4c, d show the effects of anti-TGF-β mAb treatment on AHR to MCh (**c**) and the inflammatory cell profile of the BALF (**d**) in naive HFD mice compared with naive ND mice. ^‡^*P* *<* 0.01, *P < 0.001. HFD high-fat-diet, ND normal diet, TGF transforming growth factor, H&E hematoxylin and eosin, PAS periodic acid-Schiff, MT Masson’s trichrome

Histological changes in lung tissue were evaluated by H&E, PAS, and MT staining. HFD feeding did not increase the number of inflammatory cells located in the peribronchial and perivascular areas or the number of PAS +, mucus-containing, metaplastic goblet cells. However, MT staining revealed increased peribronchial and perivascular collagen deposition in the HFD groups (Fig. 4a). To confirm this histological finding, we examined the collagen content of the lung tissue using a hydroxyproline assay, and the result was consistent with that observed by MT staining (Fig. 4b).

### Inhibition of TGF-β1 ameliorated HFD-induced AHR and fibrosis

The inhibition of TGF-β1 ameliorated HFD-induced AHR in naive mice (Fig. 4c). As previously shown, the HFD did not induce eosinophilic or neutrophilic inflammation, but did increase the number of macrophages in the BALF. Therefore, the inhibition of TGF-β1 in naive HFD mice had no effects on the inflammatory cell features of the BALF, except for the attenuation of the increased number of macrophages (Fig. 4d). We also evaluated the effects of inhibiting TGF-β1 on lung tissue histopathology in HFD-induced obesity mice. The inhibition of TGF-β1 in naive HFD mice ameliorated HFD-induced peribronchial and perivascular fibrosis (Fig. 4a). Consistently, lung tissue hydroxyproline levels were attenuated by the administration of the anti-TGF-β1 antibody (Fig. 4b). Taken together, these results indicated that the inhibition of TGF-β1 ameliorated HFD-induced fibrosis in the lungs.

### Inhibition of TGF-β1 attenuated goblet cell hyperplasia, but did not affect OVA-induced AHR and allergic inflammation

**Fig. 5:**
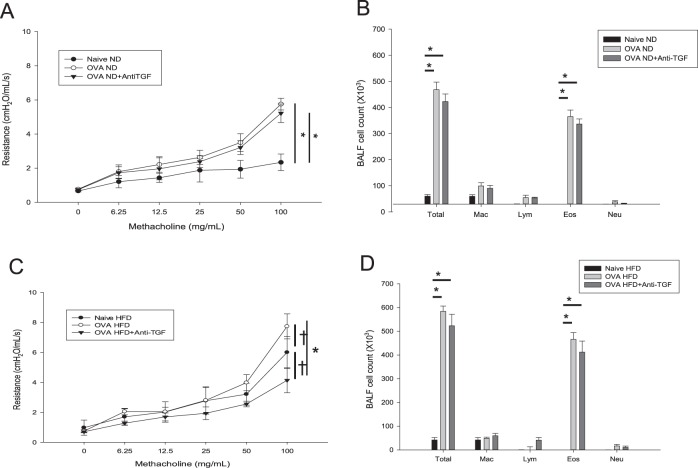
Anti-TGF-β1 treatment to ND or HFD mice with OVA allergic asthma model. Effects of anti-TGF-β mAb treatment on AHR to MCh and the inflammatory cell profile of the BALF in OVA-ND (**a**, **b**) and OVA-HFD mice (**c**, **d**). ^‡^*P* < 0.01; *P < 0.001. TGF transforming growth factor, AHR airway hyperresponsiveness, MCh methacholine, BALF bronchoalveolar lavage fluid, HFD high-fat-diet, ND normal diet, OVA ovalbumin

**Fig. 6:**
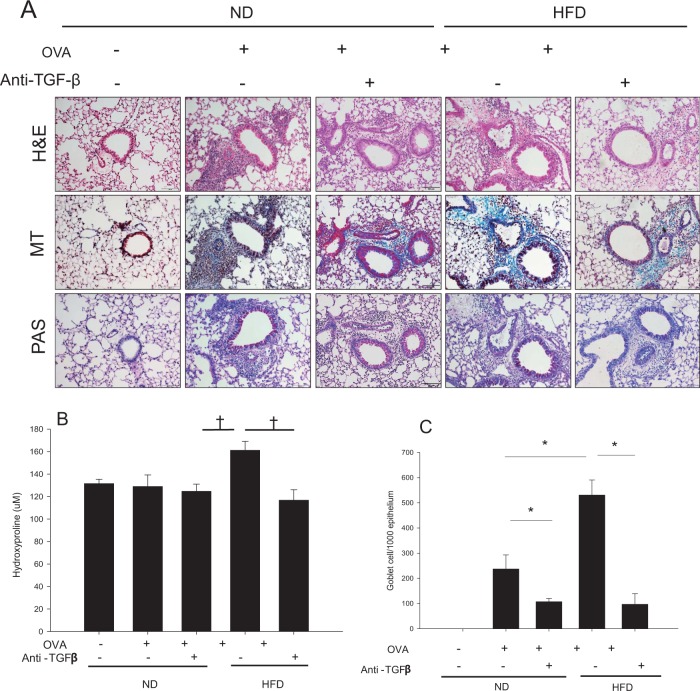
Histological features of the lung tissue from OVA-HFD and OVA-ND mice given anti-TGF-β mAb treatment. H&E, PAS, and MT staining features (**a**) are shown at ×200 magnification. The collagen content in lung homogenates was measured by a hydroxyproline assay (**b**), and the quantification of goblet cell hyperplasia measured by PAS staining (**c**) in various murine models after the inhibition of TGF-β is shown. ^†^*P* < 0.05, *P < 0.001. HFD high-fat-diet, TGF transforming growth factor, H&E hematoxylin and eosin, PAS periodic acid-Schiff, MT Masson’s trichrome

To evaluate the differences in AHR and airway inflammation induced by obesity and OVA, we examined the effects of anti-TGF-β mAb administration on the OVA-induced asthma model in ND and HFD mice (Fig. 5a–d). As expected, AHR was significantly increased in the OVA-ND mice, but treatment with the anti-TGF-β mAb did not affect AHR or the inflammatory cell profile of the BALF (Fig. 5a, b). However, the inhibition of TGF-β attenuated goblet cell hyperplasia in the OVA-ND mice (Fig. 6a–c). The OVA-HFD mice had more aggravated AHR and goblet cell hyperplasia in the epithelium compared with the naive HFD or OVA-ND mice (Figs 5c, 6c), and TGF-β1 inhibition in these mice reduced AHR but did not affect the inflammatory cell profile of the BALF (Fig. 5c, d).

Histological examination showed increased inflammatory cell infiltration in the peribronchial and perivascular areas and increased goblet cell metaplasia in the OVA-ND and OVA-HFD mice compared with the naive ND mice (Fig. 6a–c). MT staining showed minimally increased fibrosis in the OVA-ND mice, but anti-TGF-β mAb treatment did not affect the fibrosis in those mice. As expected, the OVA-HFD mice exhibited enhanced fibrosis compared with the OVA-ND mice. The OVA-HFD mice receiving anti-TGF-β mAb treatment exhibited attenuated lung fibrosis measured by the hydroxyproline assay and even attenuated goblet cell hyperplasia compared with the untreated OVA-HFD mice (Fig. 6b, c).

### In vivo intranasal administration of insulin and in vitro respiratory epithelial cell stimulation with insulin

**Fig. 7:**
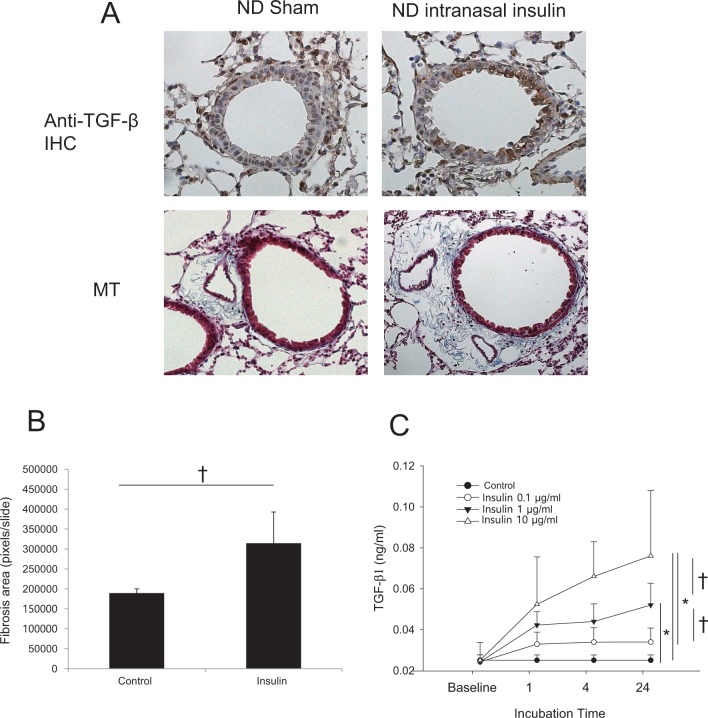
Anti-TGF-β1 immunohistochemical staining and Masson’s trichrome staining after repeated intranasal inoculations of insulin. Panel **a** is shown at ×200 magnification. The quantification of the lung fibrosis score calculated using Masson’s trichrome staining measured by Metamorph^®^ software (**b**) is also shown. The expression of TGF-β1 by BEAS-2B cells was dose-dependently increased by different concentrations of insulin for different incubation times (**c**). TGF transforming growth factor. ^†^*P* < 0.05, *P < 0.001

We hypothesized that the increased insulin exposure caused by aggravated insulin resistance in HFD mice may induce TGF-β1 excretion from the respiratory epithelium. Therefore, we intranasally inoculated ND mice once daily with 20 μg of insulin for 11 days and performed anti-TGF-β1 immunohistochemical staining and Masson’s trichrome staining on the lung tissue. As expected, we found increased TGF-β1 expression in the bronchial epithelium and peribronchial and perivascular fibrosis following the inoculation with insulin (Fig. 7a). A quantification of lung fibrosis with Metamorph^®^ software also showed lung fibrosis was enhanced by repeated insulin administrations in the ND mice (Fig. 7b). We also stimulated BEAS-2B epithelial cells with 0.1, 1, or 10 μg/ml insulin and found that insulin stimulation at the concentrations of 1 and 10 µg/ml significantly enhanced TGF-β1 expression in the bronchial epithelial cells by 24 h (Fig. 7c).

## Discussion

Obesity exacerbates asthma, and asthma patients with obesity may differ from classic asthma patients^28^. However, the mechanism underlying obesity’s contribution to asthma development has not yet been completely elucidated. In this study, we observed that HFD-induced obesity increased insulin resistance and the expression of TGF-β1 in the lungs, causing peribronchial and perivascular pulmonary fibrosis and aggravated AHR to MCh in mice.

Several studies also have shown that mice fed a HFD develop AHR and fibrosis^11,12,14,29^. As the HFD did not enhance eosinophilic or neutrophilic inflammation in the lung tissue or BALF, the mechanism of AHR in obesity seems to be quite different from that in allergic asthma. In this study, we also measured Th2 cytokine levels in the lung tissue, and the levels were not increased in naive HFD mice. These results indicated that Th2-related inflammation did not play an important role in HFD-induced lung fibrosis or AHR.

Pulmonary fibrosis is an important pathological feature of HFD-induced obesity in mice^12,20^. In this study, HFD-induced pulmonary fibrosis was confirmed by MT staining of the lung tissue and a hydroxyproline assay, and the expression of TGF-β1 was enhanced. Many studies have demonstrated that obesity itself increases lung elastance and may cause the peripheral airways to become more collapsible, thereby increasing airway resistance or functional residual volume. However, our study demonstrated that HFD-induced obesity could lead to peribronchial and perivascular fibrosis, suggesting a causal relationship between fibrosis and AHR in addition to the mechanical impairments caused by enhanced elastance. TGF-β1 signaling has been considered the major pathogenic mechanism for pulmonary fibrosis and inflammatory cell recruitment in asthma and other lung diseases^16,30,31^. TGF-β1 may be involved in the differentiation of airway smooth muscle cells^32^ and may alter the asthma phenotype, favoring a more contractile or AHR type^33^. Therefore, we investigated whether the TGF-β1 pathway played a role in HFD-induced AHR and fibrosis in mice. TGF-β1 may also enhance goblet cell hyperplasia and mucus production, which are two of the characteristic features of asthma^34,35^, and our study also showed that HFD feeding aggravated the goblet cell hyperplasia induced by OVA sensitization and challenge. However, naive HFD mice did not have increased mucus production by PAS staining, suggesting that the enhanced TGF-β1 production induced by the HFD or insulin resistance alone was not sufficient to induce goblet cell hyperplasia, but indicating the presence of the synergistic effects of TGF-β1 and Th2 cytokines or the proinflammatory milieu in asthma. Other inflammatory factors or mechanical factors in addition to enhanced insulin resistance or TGF-β1 expression may be required for the aggravation of goblet cell hyperplasia in obesity-induced asthma.

Several studies have showed that the levels of systemic inflammatory markers, such as IL-6, CRP, and TNF-α, are elevated in obesity-associated asthma^11^, and that the levels of these inflammatory markers are also dramatically decreased by bariatric surgery or weight reduction^2,4,8,10^. As previously mentioned, we also suggested that TNF-α produced by macrophages plays a crucial role in AHR development in obesity-associated asthma using C57BL/6 mice;^11^ this mechanism may involve the induction of TGF-β1 expression^36^ and insulin resistance in obesity^37^. Furthermore, a HFD alone may induce neutrophilia in the sputum, and decreased bronchodilation responses through the activation of the Toll-like receptor pathway^38^, even though we and other investigators could not find neutrophilic inflammation in an HFD-induced obesity murine model. Obesity also increases the physiological burden on respiration, and it may compress lung tissue, decreasing the functional residual capacity and total lung capacity and increasing airway resistance; these effects are more profound in asthmatic patients, as their distal lungs are more collapsible than those of normal subjects^2^. These physiological effects cause increased breathing requirements, possibly causing difficulty in the diagnosis of true asthma^39^.

We also showed that treatment with a neutralizing anti-TGF-β mAb ameliorated HFD-induced AHR, lung fibrosis, and goblet cell hyperplasia in the OVA-induced asthma model, which reflected the possible role of anti-TGF-β treatment in asthmatic patients with obesity or patients with severe asthma. Although asthma has been recognized as a reversible airway obstruction, it also has an irreversible obstructive component, especially in steroid-intractable severe asthma or asthma associated with obesity, and the prevention of irreversible airway obstruction is one of the primary goals of asthma management^40^. However, neutralizing TGF-β did not affect AHR and allergic inflammation in OVA-ND mice. These results suggest the complexity of AHR pathogenesis and support the hypothesis that anti-TGF-β1 treatment may play a significant therapeutic role, especially in asthmatic patients with obesity. However, TGF-β1 has anti-inflammatory effects, and is a key player in maintaining the balances between tolerance to self and autoimmunity or inflammatory responses to foreign antigens^41^, and some studies have reported that TGF-β1 inhibition enhances AHR and eosinophilic inflammation in an allergic asthma model^27,42^; however, our results were not consistent with this hypothesis. Thus, a careful approach is required to apply this strategy for the management of asthma.

However, the mechanism underlying obesity-induced TGF-β1 expression is not yet known. Obesity is also recognized as the stimulating factor for TGF-β1 expression through TNF-α^36^, and we already showed that obesity can enhance TNF-α production by macrophages^11^. Prior to participating in airway remodeling, latent TGF-β must be cleaved into the active form; and several factors, such as reactive oxygen species, integrins, matrix metalloproteinases, and insulin-like growth factor, have been recognized as contributors to this process^16,43^. In this study, we showed that HFD mice have significant weight gain, increased blood glucose and insulin levels, impaired glucose tolerance, and increased insulin resistance. We speculated that increased insulin like growth factor levels in HFD-induced obesity mice may also increase the conversion of latent TGF-β1 into the active form. Immunohistochemical staining of HFD mouse tissue in this study showed that the major source of TGF-β1 expression is the bronchial epithelium. Furthermore, we found that exposure to insulin could induce TGF-β1 expression in bronchial epithelial cells. Immunohistochemical studies in prior research previously showed that the bronchial epithelium and submucosal cells are the main sites of TGF-β1 production in an HFD-induced obesity model^17,27,44,45^. Singh et al. reported that insulin treatment significantly increases the proliferation and collagen production of airway smooth muscle cells through the β-catenin pathway^46^, and insulin can also induce collagen production by adipose tissue M2 macrophages via TGF-β^25^. Our data and previous studies strongly suggest that various cells may be involved in obesity-induced asthma. Hyperglycemia or hyperinsulinemia can also enhance cell–surface TGF-β receptor expression through the PI3K-Akt pathway, suggesting the possible presence of an insulin-induced autocrine TGF-β signaling pathway^23^. An interaction between TGF-β1 and insulin resistance has been reported previously. Hong et al. reported that HFD-induced TGF-β expression provokes insulin resistance in a Drosophila obesity model^21^. Those study results and our results suggest the presence of a vicious cycle between increased insulin resistance and TGF-β expression in obesity. Furthermore, hyperinsulinemia in HFD-induced obesity may also mediate bronchoconstriction through the loss of the inhibitory effect of M2 muscarinic receptor on parasympathetic nerves^47^.

This study had several limitations. First, we used male C57BL/6 mice, as weight gain is more prominent in male mice than in female mice. A prior study suggested that there is a sex-specific effect of obesity on airway inflammation, especially in adult female asthmatic patients. Scott et al. showed that the neutrophil percentage in the sputum correlated with BMI only in obese female asthmatic patients, not in male obese asthmatic patients^10^. This study did not examine the possible effect sex differences may have had on our results. Second, our research did not fully explain why severely obese subjects without insulin resistance can have uncontrolled asthma. We think another mechanism may be involved in that process.

In conclusion, HFD-induced obesity causes AHR and peribronchial and perivascular lung fibrosis through the TGF-β1 pathway, and TGF-β1 can be produced by the bronchial epithelium via stimulation with insulin. Our results suggest that insulin resistance may be the underlying cause of asthma aggravation in obesity-associated asthma and support strategies targeting insulin resistance, weight reduction, or anti-TGF-β1 management to treat uncontrolled asthma in obese patients.
